# The 5% Problem: How the Biomedical Community Responded to the Animal-to-Human Translation Crisis, and the Case for Non-Animal Methods

**DOI:** 10.3390/ani16142128

**Published:** 2026-07-09

**Authors:** Cédric Sueur

**Affiliations:** 1 UMR 7178, Institut Pluridisciplinaire Hubert Curien (IPHC), CNRS, Université de Strasbourg, 67000 Strasbourg, France; cedric.sueur@iphc.cnrs.fr; 2 Institut Universitaire de France (IUF), 75005 Paris, France

**Keywords:** animal experimentation, translational failure, new approach methodologies (NAMs), organoids, organs-on-chip, induced pluripotent stem cells, regulatory acceptance, Three Rs, replacement

## Abstract

Background: A widely cited 2024 analysis reported that only 5% of therapeutic interventions tested in animals obtain regulatory approval for human use, reviving the debate about the predictive value of animal models. This review asks how the scientific community has interpreted that finding and what collective position is emerging. Methods: The literature citing the original study was assembled from public citation indexes and classified by argumentative stance into five categories: defences of animal experimentation, critiques, methodological and disease-specific analyses, the author’s own position, and work developing non-animal methods; no new animal data were generated. Results: Across the corpus, attempts to strengthen the validity of animal studies tended to reduce rather than reinforce their apparent translational signal; the low translation rate recurred across unrelated disease domains; and even defenders of animal models increasingly restricted their claims to specific contexts while conceding poor translation. In parallel, non-animal methods—including organoids, organ-on-chip systems, and computational models—were repeatedly described as equalling or surpassing animal models in several fields. Conclusions: The scientific, ethical, economic and regulatory cases now converge, supporting a deliberately planned transition toward human-relevant methods rather than its deferral.

## Simple Summary

For more than a century, new medicines have first been tested on animals before being tried in people, yet a recent large study found that only about one in twenty therapies that work in animals is later approved for human use. This review gathered and examined the scientific articles that responded to that finding, to understand how researchers now view the usefulness of animal experiments. Three patterns emerged. First, making animal studies more careful and rigorous tends to show that their results predict human outcomes even less well than was thought. Second, the same low success rate appears again and again across very different diseases. Third, methods that do not use animals—such as human cells grown in a laboratory, miniature human tissues, and computer models—are now good enough to replace animal testing in a growing number of areas. Taken together, the scientific, ethical, economic and regulatory reasons to move away from animal testing now point in the same direction. The main message is that this change is already underway, and that planning it carefully would benefit both human health and animal welfare.

## 1. Introduction

The use of animals to predict human biological responses is one of the foundational assumptions of modern biomedical research. It is also one of the least examined empirically. In 2024, Ineichen et al. addressed this question directly: pooling data across therapeutic areas, they found that, of the interventions that succeed in animal studies, only around 5% go on to obtain regulatory approval for human use [1]. The same group has separately formalised the methodology of preclinical systematic reviews, underlining how much of the evidence base rests on heterogeneous and often poorly reported animal studies [2]. The 5% figure is not, in itself, novel—attrition in drug development has been discussed for decades—but its provenance from a transparent, systematic analysis gave it unusual force, and the biomedical community responded at scale.

That response has been neither uniform nor easily summarised. Some authors defend animal models as indispensable in specific domains; others treat the 5% figure as confirmation that the paradigm is scientifically exhausted and ethically untenable; many cite the finding in passing while continuing to rely on animal experiments; and a growing number use it to motivate the development of human-relevant alternatives. The purpose of this article is to map that response rather than to assert a false consensus. We take the population of works that cite Ineichen et al. [1] as a natural sample of how the field is reacting, and we ask a simple question of each: what position does it take on the continued use of animals in biomedical research?

**Figure 1:**
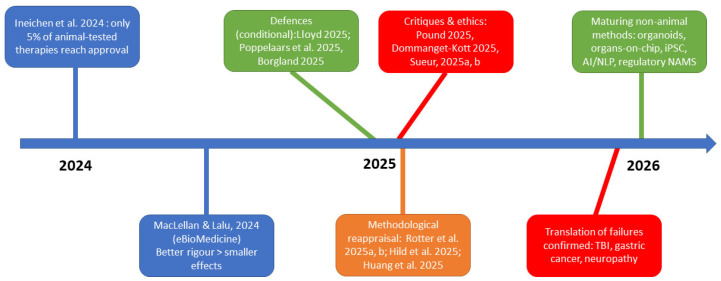
The post-2024 debate on animal-to-human translation. Following the report by Ineichen et al. [1] that only ~5% of animal-tested therapeutic interventions reach regulatory approval, the literature of 2024–2026 fans out into four strands. Conditional defences of animal models (Lloyd [3]; Poppelaars et al. [4]; Borgland [5]) are set against scientific and ethical critiques (Pound [6]; Dommanget-Kott [7]; Sueur [8,9]) and against methodological reappraisals showing that greater rigour tends to yield smaller effect sizes (MacLellan & Lalu [10]; Rotter et al. [11,12]; Hild et al. [13]; Huang et al. [14]). Disease-specific translation failures are confirmed in traumatic brain injury [15], gastric cancer [16] and diabetic peripheral neuropathy [17], while a rapidly maturing ecosystem of non-animal methods—organoids, organs-on-chip, iPSC systems, AI/NLP approaches and regulatory NAMs—takes shape [18,19].

To do so, I retrieved the records that Google Scholar indexes as citing the original study (89 records at the time of analysis). After consolidating three duplicate deposits—two language versions of one review, and two repository deposits of two further works—86 distinct works remained. We read each and assigned it to one of five positions: (i) defences of animal experimentation; (ii) critiques of animal experimentation; (iii) other and methodological perspectives, which also encompasses the large group of domain reviews that invoke the 5% figure as context; (iv) the present author’s own published position; and (v) contributions oriented toward non-animal methods. The categories are mutually exclusive and exhaustive, so that the sub-totals sum to the corpus. This is a narrative and interpretive review, not a quantitative bibliometric study; the value of the classification lies in revealing the shape of the debate, summarised in Figure 1.

## 2. Methods

### 2.1. Sampling Strategy and Search

This review used a citation-based sampling strategy rather than a keyword search. Instead of querying a research question directly, the corpus was defined as the population of publications indexed as citing a single index study; Ineichen et al. [1], whose finding—that only 5% of animal-tested therapeutic interventions obtain regulatory approval for human use—is the specific object of this review. This approach was chosen to capture the field’s response to a defined empirical claim more directly than a topic-based search would; its main limitation, discussed below, is that it excludes works that engage with the same debate without citing [1].

Because the sampling unit was a citation relationship rather than a set of query terms, the search strategy consisted of citation tracking rather than a Boolean expression. On 21 June 2026, the indexed record for Ineichen et al. [1] was located in Google Scholar and every item listed under its “Cited by” link was retrieved and exported, returning 89 records. Google Scholar does not expose an exportable keyword string for cited-by tracking, and its citation index is dynamic; the retrieval is therefore defined by the index study and the retrieval date, and the record count is reported as of that date and may differ on later re-querying. Google Scholar was used as the single source because its cited-by index is broader than that of Scopus or Web of Science and thus better suited to the review’s aim of capturing the widest possible range of responses, including grey literature and non-indexed venues; the trade-off is the lower curation and reduced reproducibility just noted, which we accept as a deliberate feature of a narrative rather than systematic design.

### 2.2. Inclusion Criteria and Record Types

Records were screened for duplication rather than topical relevance. Three duplicate deposits were identified and consolidated—two language versions of a single review, and two separate repository deposits of two further works—yielding 86 distinct records. No relevance-, language-, date-, venue- or quality-based exclusion criterion was applied: because the aim of the review is to map the full range of the field’s response to [1], including tangential, low-quality, or non-scientific citations, all 86 distinct records were retained for classification rather than filtered out. Deduplication was thus the only exclusion rule applied. This is a deliberate departure from standard systematic-review screening and is stated explicitly here to distinguish this narrative review from a systematic one.

Record type was recorded but never used as an inclusion or exclusion criterion. Preprints were retained alongside peer-reviewed articles and flagged as such (e.g., [14,20,21]), on the grounds that they form part of the field’s real-time response to [1]; where a preprint had since been published, the peer-reviewed version was consulted. Non-article records were treated identically: books and book chapters (e.g., [8]), editorials, commentaries and short opinion pieces (e.g., [3,22]), theses, reports, and even a single non-scientific news item of doubtful reliability [23] were all classified by argumentative stance in the same way as research articles. Retaining these record types is intentional and is itself part of the finding, since it documents how far the 5% figure has diffused beyond the primary research literature; their limited analytical weight is discussed in Section 6.3.

### 2.3. Classification

Each of the 86 records was read in full by the author (C.S.) and assigned to exactly one of five predefined, mutually exclusive and exhaustive stance categories: (i) defences of animal experimentation; (ii) critiques of animal experimentation; (iii) methodological and other perspectives, encompassing domain reviews that cite [1] chiefly as contextual framing; (iv) the author’s own previously published position; or (v) contributions developing or advocating non-animal methods (NAMs). Assignment was based on the position each work takes on the continued use of animal models, not on its disciplinary field.

All screening and classification were performed by a single reviewer (C.S.); no second coder was involved and no inter-rater reliability statistic is therefore available, which is acknowledged as a limitation and should be weighed when interpreting the sub-totals. To mitigate the subjectivity this entails, the classification rule was fixed in advance, the categories were defined to be mutually exclusive and exhaustive, and each record was read in full rather than screened by title and abstract alone. The author has himself published work classified under category (iv) [8,9]; this is disclosed in the Conflicts of Interest statement. No new animal data were generated or analysed for this review.

## 3. The Translation Failure: Scope and Consistency

Before turning to the positions taken in the corpus, it is worth establishing what is, and is not, contested. One clarification is needed before proceeding, because the 5% figure is open to over-reading. Regulatory approval is a multi-causal endpoint: an intervention can fail to reach it for reasons unrelated to the predictive validity of animal models—unfavourable commercial viability, insufficient funding, manufacturing or formulation difficulties, or weaknesses in trial design—and attrition arising from these causes is not, in itself, evidence that an animal model mispredicted a human response. The 5% figure should therefore not be read as a direct or exclusive measure of animal-model failure. Two considerations nonetheless preserve its relevance here. First, the causal categories are not all external to animal-model predictivity: insofar as an intervention fails through lack of efficacy or unanticipated toxicity in humans, that failure is itself translational, because in each case an animal model had indicated an efficacy or a safety profile that did not hold in people; such attrition is a manifestation of poor animal-to-human prediction rather than a confounder of it. Second, and more decisively, the argument developed in this review does not rest on the 5% figure alone. The other two lines of evidence assembled below are independent of regulatory endpoints: the finding that strengthening the rigour and external validity of animal studies shrinks rather than reinforces their effect sizes [10,11,14] concerns the reliability of the preclinical signal itself, not drug approval; furthermore, the disease-specific failures documented across mechanistically unrelated domains [15,16,17,24] are failures of efficacy and mechanism in humans, not commercial or manufacturing withdrawals. The convergent case therefore holds even if regulatory attrition is discounted as multi-causal. The empirical claim that animal-to-human translation is weak is corroborated well beyond the original report. A particularly instructive result predates it: MacLellan and Lalu showed that when animal studies are conducted with enhanced external validity, effect sizes do not grow more reliable—they shrink, by an amount consistent with the removal of bias from less rigorous work [10]. This is the inverse of what one would expect if poor translation were merely a quality problem solvable by better animal studies.

The pattern recurs across disease areas. In traumatic brain injury, Dobson et al. note that despite decades of high-quality animal research, no pharmacological intervention has reduced human mortality [15]. In oncology, Shyu et al. document that several gastric-cancer therapies effective against animal models—EGFR, MET, FGFR2 and PI3K inhibitors among them—subsequently failed to improve human outcomes [16]. In diabetic peripheral neuropathy, Liu and colleagues describe paeoniflorin as effective in animal models yet stalled by exposure and translational barriers in humans [17]. Reviewing the broader picture, Levchenko et al. ask why, despite advances in genomics, automation and artificial intelligence, do only a minority of drug candidates entering human testing ever reach patients, and locate the root cause upstream, in the preclinical model itself [24]. The consistency of these failures across mechanistically unrelated domains is the central fact that the rest of the literature must accommodate.

Three qualifications refine this picture without overturning it. The first concerns what the 5% figure does and does not measure. It is conditional on prior success in animal studies [1]— the fraction of animal-validated interventions that reach human approval—and those interventions are themselves the output of a multi-stage preclinical pipeline, in which conventional two-dimensional cell screens (often human lines such as HeLa or Caco-2) feed compounds into whole-organism testing. The measured attrition is thus a property of the pipeline as a whole, not a verdict on animal models considered in isolation. Far from weakening the case for human-relevant methods, this strengthens it: inserting more predictive in vitro and in silico systems—organoids, organ-on-chip devices and computational models—earlier in the process should enrich the pool of genuinely translatable candidates and therefore raise, rather than merely relocate, the downstream success rate [18,25,26]. Replacing the least human-relevant links of the current pipeline is exactly what this review advocates.

The second qualification is that part of the measured gap may reflect how well models are constructed, resourced and reported rather than an irreducible divergence between species. The quality and completeness of preclinical evidence are demonstrably uneven [2,13,27], and the infrastructure that supports animal work—validated antibodies, standardised reagents and protocols—is less developed for some species than for the standard laboratory rodent. The data nonetheless bound how much of the gap this can explain: were under-resourcing the dominant cause, more rigorous animal studies should translate better, whereas enhancing their rigour and external validity tends instead to shrink their apparent translational signal [10,11,27]. A real animal-to-human gap therefore persists once model-quality factors are set aside, even though those factors merit attention in their own right.

The third qualification is that “animal models” is not a single entity. Predictive value varies markedly with species, strain and model design [20,28], and several familiar limitations are model-specific rather than universal: immunodeficient transgenic mice lack a competent immune system, and laboratory rodents seldom develop the spontaneous tumours that arise in species such as the dog. These contrasts cut both ways. They warn against applying one translation rate uniformly across all animal research, and they mark out higher-value niches—notably naturally occurring disease in companion animals, which sustains genuine veterinary clinical trials with reverse-translational benefit to the animal patients themselves [29,30]—where in vivo data remain, for now, hard to obtain otherwise. Tellingly, NAMs are already closing some of the gaps that once justified such models: immune competence, long a weakness of in vitro systems, is now being engineered into organ-on-chip platforms [19].

## 4. Defences of Animal Experimentation

Ten works in the corpus defend the continued use of animal models. What is striking is that none defends the status quo unconditionally. Lloyd makes the argument most explicitly: simply ending animal testing, he contends, is not the answer, because alternatives are not yet validated for every purpose; the remedy is better science—pre-registration, transparent reporting, and strategic use of animals alongside human methods—rather than abolition [3]. Poppelaars et al. defend animal models in the specific context of complement immunology, where, they argue, whole-organism complexity has been essential to clinical breakthroughs [4]. Borgland advances a parallel case for psychiatry, holding that the heterogeneous and circuit-level mechanisms of mental illness are still best probed in animals even when effect sizes translate imperfectly [5]. Banerjee and colleagues frame the diversity of neuroscience model organisms itself as a scientific asset rather than a liability [28].

A second cluster of defences comes from critical-care and intensive-care medicine, where systemic, multi-organ pathophysiology is difficult to reconstruct in vitro. Fraser and colleagues review the current reality of animal models of critical illness and argue for their continued, if reformed, use [31]; Fujinami et al. extend this assessment to the Asia-Pacific region, documenting shared challenges and future directions [32]. Others propose improving the models rather than replacing them: Vera-Tizatl and colleagues introduce the Vietnamese swine as a translational model of breast carcinoma [33]; Schachtschneider et al. describe a next-generation large-animal oncology platform explicitly designed to incorporate new approach methodologies and to anticipate regulatory change [34]; and Ball and colleagues offer a data-driven tool for prioritising mouse strains to improve preclinical modelling [20]. Popiel-Dziewierz and colleagues, finally, highlight reverse translation in veterinary cardiology, where procedures first tested in animals benefit the animal patients themselves [29].

The common structure of these contributions is important. Each restricts its claim to a domain, concedes the reality of translation failure, and frames animal use as conditional, reformable, or transitional. This is a marked departure from earlier defences that treated animal models as broadly and reliably predictive, and it narrows the practical distance between defenders and critics more than the rhetoric suggests.

## 5. Critiques of Animal Experimentation

Twelve works mount explicit critiques, on scientific or ethical grounds, or both. The scientific critiques largely overlap with the failure cases already discussed—Dobson et al. on traumatic brain injury [15], Shyu et al. on gastric cancer [16], Liu et al. on diabetic neuropathy [17], and Levchenko et al. on the systemic root of attrition [24]—but several authors go further and locate the problem in the explanatory logic of the model rather than in any particular result. Pinto-Ribeiro, reviewing animal models of traumatic neuropathic pain, argues that decades of comfortable methodological freedom have produced mechanisms that are frequently species-specific or irrelevant to human psychology, so that the emotional and affective dimensions of pain remain poorly captured [35].

The ethical and institutional critiques are at least as forceful. Lewis documents that learners increasingly object to educational activities involving animals, and that regulators and funders are actively driving reduction and replacement in teaching [36]. Pound, drawing on Bourdieu, offers a sociological account of why non-animal methods are being phased in without a corresponding decrease in animal use: the field’s incentive structures—funding priorities, career paths, journal metrics—make individual transition costly even when the science supports it [6]. Dommanget-Kott examines the ethics directly, arguing that the ‘greater good’ justification for animal suffering is empirically undermined once translation rates are taken seriously [7]. Manti reaches a similar conclusion from a different starting point, noting that the FDA Modernization Act 2.0 removed the statutory requirement for animal testing and inferring that such testing was never a scientific necessity [37]. Fluck and Zillmann add a forward-looking ethical dimension, asking how the emergence of human cerebral organoids reshapes the question of the moral status of laboratory animals [38].

Two further works document the institutional reality in concrete terms. Cash and Courtot investigate the publication rate of recent non-human-primate projects in France and find it low relative to the ethical cost incurred [39], while Rafi and colleagues—surveying animal models in molecular biology—frame the ethical imperative and the path to human-relevant translation as inseparable [40]. Taken together, the critical literature converges on a single proposition: that the limits of animal models are not an incidental quality problem but a structural one, with ethical consequences that the translation data make harder to set aside.

## 6. Methodological and Other Perspectives

The largest group in the corpus—39 works—neither defends nor rejects animal research outright. It comprises, first, a methodological literature that scrutinises how preclinical evidence is produced and appraised, and second, a broad and heterogeneous set of domain reviews that cite the 5% finding chiefly as context. Both are informative about the state of the field, but in different ways.

### 6.1. The Methodological Literature

Several works examine the machinery of preclinical evidence itself. Hild and colleagues conduct an umbrella review of animal systematic reviews and find their quality and scope highly variable [13]. Rotter and colleagues report that confirmatory multi-laboratory studies, intended to strengthen translation, tend to deflate effect sizes once between-laboratory variability is properly accounted for [11], a finding echoed in their companion synthesis of stakeholder discussions [12]; this complements the enhanced-validity result of MacLellan and Lalu [10]. Huang and colleagues evaluate, by simulation, whether replication-success metrics borrowed from other fields apply to animal-to-human translation, and caution that they tend to overestimate predictive value [14]. Liesner et al. assess how rigorously systematic-review methodology is actually used in EFSA opinions on animal health and welfare, exposing inconsistent reporting [27]. Gallas-Lopes and colleagues provide a worked example in their meta-analysis of NMDA antagonists and social behaviour, where preclinical heterogeneity is substantial [41]. The cumulative message of this literature is the paradox at the centre of the debate: improving the rigour of animal studies tends to reveal smaller, not larger, translational signals.

### 6.2. Comparative and Bridging Studies

A further set of works sit at the animal–human interface without taking a strong side. Chong Chie and colleagues directly compare preclinical and clinical findings in Alzheimer’s disease, mapping where the two diverge [42]. Zhuang et al., working on pig lung transplantation, explicitly flag interspecies physiological disparities as a limitation of their own small-animal-derived inferences [43]. Nieto and Ray integrate preclinical, human-laboratory and clinical evidence in alcohol use disorder, modelling a more human-anchored development pathway [44]. Robinson and colleagues offer a practical framework for launching translational clinical trials [45], and Mälberg’s thesis on cardiopulmonary resuscitation combines experimental and clinical insights [46]. Two contributions add system-level context: Yang situates the translation problem within the geopolitics of biotechnology competition [22], and Cicchelero and De Rooster discuss clinical trials in companion animals as an alternative source of naturally occurring disease data [30].

### 6.3. The Wider Citation Footprint

The remainder of this group illustrates how widely the 5% figure has diffused. A large number of domain reviews invoke it as a framing statistic while continuing to rely on, or simply to describe, animal-based work. These span natural-product and antioxidant pharmacology [47,48], psychiatric and metabolic pharmacology [49,50,51], cardiac and cardiovascular research [52,53], nutrition and food science [54], microbiome and infection studies [55,56,57,58], oncology and toxicology [59,60,61], wound healing and regenerative contexts [62], anti-ageing pharmacology [63], and a range of human-cohort and observational studies that bypass animals entirely [64,65,66]. Several are only loosely connected to the translation question—including studies in rats on intestinal gene expression [67], a pregnancy nutrition pilot trial [68], and a human single-cell skin-disease analysis [69]—and at least one indexed record is a non-scientific item of doubtful relevance and misinformation [23], a reminder that a raw citation count is a coarse instrument. We include these works for completeness and transparency, but their analytical weight is limited: most cite Ineichen et al. [1] as a rhetorical anchor rather than engaging with its argument. Their very heterogeneity, however, is itself a finding—the 5% result has become a shared point of reference across biomedicine, even where the citing work does not act on it.

## 7. The Author’s Position

My own contribution to this debate is first methodological before it is ethical. In a 2025 analysis, I re-examined the concordance statistics underlying claims of strong animal-to-human agreement and argued that the frequently cited figure of roughly 86% concordance is inflated by selective reporting and the file-drawer effect; once these are taken into account, the true translation rate is plausibly at or below the 5% threshold rather than above it [9]. The methodological point matters because the defence of animal research often rests on an optimistic reading of concordance that the data do not support.

The ethical and conceptual argument is developed at greater length elsewhere. In *Décoloniser notre rapport aux animaux* (decolonising our bond with animals) I argue that the central question is not whether we may use animals but why we have organised knowledge production around their exploitation in the first place, and that a more honest reckoning with sentience and with the interests of other animals reshapes what counts as legitimate science [8]. This position is consistent with the broader assessment of the field offered by the recent CNRS prospective report on the animal condition in the social sciences and humanities, to which colleagues (but not I) contributed, which documents the maturation of animal studies as a research domain in France [70]. My position, in short, is that the scientific case and the ethical case point the same way: the weakness of translation removes the principal utilitarian justification for animal experimentation, leaving the ethical objection without a countervailing benefit large enough to outweigh it.

## 8. Toward Non-Animal Methods

Twenty-two works in the corpus develop or advocate non-animal, human-relevant methods—collectively termed new approach methodologies (NAMs). A terminological distinction should be made explicit here, because the label “non-animal” can obscure it. The translational critique developed in the preceding sections concerns in vivo whole-organism experimentation—the use of living animals to predict human responses—and not the use of animal-derived biological material as such; the two are not equivalent. Several widely used in vitro systems rest on immortalised animal cell lines: the canine MDCK line, for example, serves for epithelial-barrier and -transport studies much as the human Caco-2 line does, and a growing number of organoid platforms are established from companion-animal or livestock tissue, including canine and porcine systems. Such models reduce, and can ultimately replace, in vivo procedures even though they originate, in part, from animal material; within the definitional boundaries now used for complex in vitro models, they are appropriately counted among NAMs [18]. Throughout this review, therefore, “non-animal” denotes the replacement of predictive in vivo animal experimentation, not the elimination of every animal-derived reagent from the laboratory. The most important point about this literature is that it no longer reads as aspirational. Mottet and colleagues provide a comprehensive review of organoids, organs-on-chip, and complex in vitro models, with explicit attention to definitions, applications, validation and ethics [18]. Within this ecosystem, several platforms now outperform animal models for specific purposes: Heiduschka and Prigione show that induced pluripotent stem cell (iPSC) models capture mitochondrial disease in human tissue that animal models reach poorly [71]; and Lessi, Caporale and Testa demonstrate that single-cell resolution exposes human neural heterogeneity that animal models cannot represent [72].

In vitro human systems are diversifying rapidly. Heikamp and colleagues model thyroid-hormone transport across the human brain barrier [73]; Joukhdar and colleagues engineer human models of biological ageing [74]; Soares and colleagues embed immune competence into organ-on-chip systems, addressing one of the field’s main historical gaps [19]; Wirsig and Bernhardt use a human multi-cell bone culture to dissect osteogenic responses [75]. Microfluidic and organ-on-chip systems now span a widening range of applications, although their maturity varies considerably by field: vascularised tumour-on-chip platforms [76], brain organoids for neurodegeneration [77], microfluidic inflammation chips [78], human cardiac models [79] and tumour vessel-on-chip devices for studying nanoparticle delivery [80] are all represented. In oncology in particular, on-chip modelling remains an area of active development rather than a settled technology: faithfully reproducing intratumoural clonal heterogeneity; sustaining stable multi-cell co-cultures; and optimising perfusion, flow and shear-stress conditions are recognised challenges that are still being resolved. Computational and artificial-intelligence methods complete the picture: Demyashkin and colleagues review AI across the drug-development lifecycle [25]; Si and colleagues present an explainable AI model for predicting synergistic cancer therapies [26]; and natural-language-processing approaches—an annotated preclinical corpus [81] and a large-scale assessment of animal-to-human translation [21]—begin to quantify translation at the scale of the whole literature. Digital-twin brain models extend the same logic to neuropsychiatry, in methamphetamine abstinence [82] and tinnitus [83].

Crucially, the regulatory environment is moving with the science rather than lagging behind it. Courtot and colleagues report on panel discussions concerning the global regulatory acceptance and harmonisation of non-animal NAMs, documenting concrete engagement by major agencies [84]; Park analyses the strategic niche that alternative testing methods now occupy in regulatory science [85]; and Daou’s work on in vitro biophysical stimulation illustrates the methodological depth now available even for complex tissue-level questions [86]. More speculative directions, such as bionic and biohybrid systems, are also emerging [87]. The combined effect is that the standard objection to replacement—that the alternatives are not ready—is increasingly difficult to sustain across the domains represented here.

## 9. A Roadmap for Transition (2026–2040)

**Figure 2:**
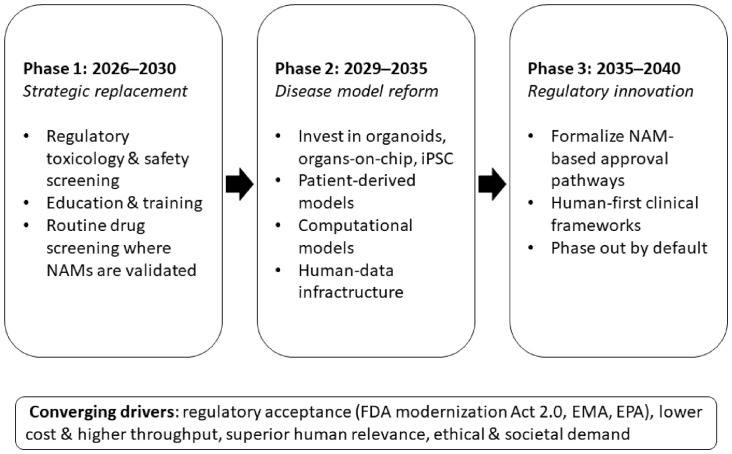
A phased roadmap for the transition to non-animal methods (2026–2040). The transition is driven by four converging forces: regulatory acceptance (including the FDA Modernization Act 2.0, and engagement by the EMA and EPA), lower cost and higher throughput, superior human relevance, and growing ethical and societal demand.

If the scientific obstacle to replacement has largely fallen, what remains is institutional. I therefore propose a phased transition rather than an abrupt prohibition, designed to align with the pace at which NAMs are validated domain by domain (Figure 2). Phase 1 (2026–2030) would prioritise strategic replacement where alternatives are already validated: regulatory toxicology and safety screening, education and training, and routine drug screening. Phase 2 (2029–2035) would reform disease modelling itself, through sustained investment in organoids, organs-on-chip, iPSC and patient-derived systems, computational models, and the human-data infrastructure on which they depend. Phase 3 (2035–2040) would consolidate the shift at the level of policy, formalising NAM-based approval pathways and human-first clinical frameworks, so that non-animal methods become the default rather than the exception. Framed in this way, the earliest phase is governed by reduction rather than immediate abolition, and it does not require abandoning animal use wherever a clear, presently irreplaceable advantage exists. Some spontaneous animal diseases occur at an incidence well above that of their human counterparts—canine hyperadrenocorticism (Cushing’s disease) being a frequently cited example—so that naturally occurring disease in companion animals can yield clinically informative data that no current in vitro system reproduces, while the affected animals are themselves candidates for the therapies tested [29,30]. Retaining such uses in the near term, where the advantage is genuine and the alternative not yet validated, is consistent with—not contrary to—the trajectory proposed here—strategic, selective animal use is precisely the content of Phase 1, and its scope is expected to contract as human-relevant models mature.

Four drivers make this trajectory plausible. First, regulatory agencies increasingly permit NAM-based data, as the panel discussions reported by Courtot et al. [84] and the statutory change noted by Manti [37] illustrate. Second, NAMs are frequently faster and cheaper than animal studies once established. Third, the ethical case—made forcefully by Pound [6], Dommanget-Kott [7] and others [88,89]—has moved from the margins to the centre of research-policy discussion. Fourth, and decisively, the human relevance of the best NAMs now exceeds that of the animal models they would replace [71,72]. None of these drivers is sufficient alone; together they make the direction of travel hard to reverse.

## 10. Conclusions

The 5% translation rate reported by Ineichen et al. [1] is not a problem that can be solved from within the animal-testing paradigm. The methodological literature shows that improving the rigour of animal studies tends to shrink, not strengthen, their translational signal [10,11,14]; the disease-specific literature shows the failure recurring across unrelated domains [15,16,17,24]; and even the defenders of animal models now restrict their claims to particular contexts and concede the reality of poor translation [3,4,5,31]. Across the corpus, the centre of gravity has shifted from whether animal models predict human responses to how quickly human-relevant methods can take their place.

Read as a whole, the literature that has formed around the 5% finding describes a field in transition. The science of non-animal methods is no longer the limiting factor; the alternatives are, in several domains, demonstrably superior, and the regulatory architecture is adapting. What remains is institutional and political: whether research systems will lead the transition deliberately or be compelled to it by regulators and public expectation. Crucially, the ethical dimension of this transition no longer rests on welfare considerations alone: a substantial and growing body of comparative evidence now establishes that the animals most widely used in research are sentient beings with genuine emotional and cognitive capacities—affective states, pain, and, in mammals and birds, forms of conscious experience [90,91,92]—and once these capacities are acknowledged, continuing to privilege human benefit over animal interests while increasingly capable human-relevant methods exist is not a scientific necessity but a form of speciesism [93,94,95]. In reaching this convergent conclusion we have sought to represent the opposing case at its strongest rather than to set it aside. The most substantial argument for retaining animal models is genuine and is advanced by serious investigators in this corpus: certain systemic, multi-organ and circuit-level pathologies—in critical-care medicine, complement immunology and psychiatry among them—are still only imperfectly reconstructed in vitro, and in these domains, whole-organism experimentation retains real, if narrowing, explanatory value [4,5,31]. A balanced reading must grant this, and Section 4 was written to give these defences their full force rather than a token hearing. What a balanced reading need not grant is that fairness to both positions requires a neutral verdict. The direction in which this review leans is not imposed on the corpus but drawn from it: the defenders surveyed here themselves restrict their claims to specific contexts and concede the reality of poor translation [3,4,5,31], while two further and independent bodies of evidence—the methodological finding that greater rigour shrinks the translational signal [10,11,14] and the disease-specific failures recurring across unrelated domains [15,16,17,24]—point the same way. Objectivity in a narrative review lies in reporting that convergence accurately, including the domains where it does not yet hold, rather than in manufacturing a symmetry the evidence does not support. The argument of this review is that the scientific, ethical, economic and regulatory cases now converge, and that the responsible course is to plan the transition rather than to defer it.

## Data Availability

The corpus analysed in this review consists of the publicly indexed records citing reference [1]; the full classified list is provided in the References section.
